# Leveraging industrial-technological innovation to achieve sustainable development: A systems thinking perspective

**DOI:** 10.1371/journal.pone.0242981

**Published:** 2020-12-21

**Authors:** Jin Guo, Meng Chen, Xialing Sun, Zhanzhao Wang, Jinli Xue

**Affiliations:** 1 School of Surveying and Land Information Engineering, Henan Polytechnic University, Jiaozuo, China; 2 Shandong Geological Environmental Monitoring Station, Jinan, China; 3 School of Management, China University of Mining and Technology, Beijing, China; 4 Research Center for Energy Economics, School of Business Administration, Henan Polytechnic University, Jiaozuo, China; Institute for Advanced Sustainability Studies, GERMANY

## Abstract

Industrial-technological innovation (ITI) has become an important requirement for the sustainable development of China. ITI development requires a comprehensive understanding of the dynamic complexity associated with ITI systems. Previous research into ITI systems is based primarily on static methods that isolate system components, and ignore feedback on adjustments made. Based on systems thinking, this paper develop six archetypes (“Limit to Growth,” “Success to the Successful,” “Tragedy of the Commons,” “Fixes that Fail,” “Accidental Adversaries,” and “Shifting the Burden”) and an ITI system integration model. The model visualizes the ITI system as a whole and identifies bottlenecks that may affect ITI development. This conceptual model provides a more effective method of judgment, which can better explain the operational mechanism of the ITI system and improve the system’s operational characteristics. Finally, we evaluate the ITI system and propose that self-organization is a key lever of a systemic intervention framework for ITI.

## Introduction

Industrial-technological innovation (ITI) is an important engine to promote China’s economic transformation and upgrading, and also a supporting force for China to build an innovative country [1,2]. The weak link of China’s scientific and technological innovation is constantly undermining China’s economic development, especially in the critical current period of China’s reform and development [3,4]. Although China has made great achievements in ITI, some Chinese industries still lack core technologies [5,6]. In recent years, development in communications, big data, high-precision instruments, and other leading industries in China has faced lack core technologies, which is also the main factor causing the inefficient use of resources in China [7]. In 2016, China’s energy consumption per unit of GDP was 5% lower than in 2012, but it was still 3.81 times that of the United States and 7.18 times that of the UK [8]. China’s annual pollutant emissions are also increasing year by year, which is closely related to the slow development of industrial technology [9,10]. To tackle this current situation, China has launched the “Made in China 2025” strategic plan, implemented an innovation drive, and created “Innovative China” to progress toward achieving sustainable development [11].

To develop sustainably, China needs to not only improve its urban environment but also change its economic and technological environment [12]. ITI is the result of internal and external factors, including coordination and evolution between society, economy, and environment [13,14]. A large number of innovations generated by technological breakthroughs promote the high-speed operation of ITI system; changes in management measures and policies related to technological innovation and changes in managers’ capabilities can all contribute to the dynamic evolution of ITI. In addition, ITI also involves many aspects. If the allocation of resources inside and outside the system is imbalanced, that affects the rate of innovation [15–17]. Moreover, constructing an innovation diffusion mechanism also requires a lot of resources. ITI is also impacted by economic structures [18], industrial transfer [19], political relationships [20], and market demand or other aspects [21], and the combination of all these factors means that ITI systems are inherently complex.

Existing research on ITI systems focuses on the development of influencing factors under static conditions. Wisdom [22] examined the relationship between government departments and ITI, finding that different types of participants in the intermediaries have different requirements for innovation, and that policy makers can positively or negatively influence innovation through intermediaries. Sanjesh [23] studied the diffusion of ITI based on the spatial distance perspective. The main finding was that ITI diffusion between regions weakens as spatial distance increases, although national imitation and adoption capabilities can lessen this impact. Li [24] studied the data of 30 projects and found that ITI reduces industrial energy consumption and pollutant emissions, and provides a basic guarantee for sustainable industrial development. Other studies have examined the static impact of factors such as optimizing resource allocation [25], increasing innovation input [26], implementing scientific management [27], and integrating innovation clusters [28]. However, the literature has ignored the dynamic correlations and systematic relationships between related factors.

The ITI system features many influencing factors, highly complex relationships, diversified development paths, and uncertainties [29]. The feedback adjustment mechanism runs through the whole system and has an important impact on the direction of the system’s evolution. To explain the system development path of ITI from a dynamic perspective, we will address the following three questions:

1. What are the different paths and obstacles in the evolution of the system?
2. What are the leverage solutions for system operational barriers?
3. What are the key influencing factors of ITI under the dynamic perspective?

This study will based on the dynamic systems perspective, explore the impact of uncertainty on the system operation process, and identify the driving factors of the dynamic perspective. It will then use system archetype theory to describe the advantages of system complexity, build a system prototype of various factors in ITI, and propose leverage solutions to break the bottlenecks. This study makes three contributions to the literature. First, it deepens research on ITI and helps to build a theoretical framework for researching the interconnections of technical innovation, ITI, and sustainable development. Second, based on the dynamic perspective, it analyzes the operation mechanism and development bottlenecks of the ITI system and seeks leverage solutions to break the bottlenecks. Finally, it extends the method of researching ITI from statistical and mathematical modeling to system archetypes, which has important reference significance for understanding and exploring China’s ITI.

## Literature review

### Conceptualization of ITI

Schumpeter’s innovation theory put forward that “innovation is the essence of development,” which has been widely accepted [30]. However, the adoption of a new method by a single enterprise cannot cause innovation in the entire industry [31]. Therefore, to achieve ITI, not only “innovation” but also “innovation expansion” must be initiated under certain conditions [32].

“Innovation” is decomposed into product innovation, technological innovation, market innovation, resource allocation innovation, and organizational innovation in Schumpeter’s innovation theory [30]. Technological innovation is the use of a new production method, which does not need to be based on new scientific discoveries or to have been empirically verified by the relevant manufacturing department [33]. Later scholars have asserted that technological innovation is the process of integrating and learning different knowledge structures and of integrating knowledge into new products [34]. Some scholars have also proposed that technological innovation is a means to improve product quality and service efficiency by introducing new processes and technologies [35].

To fully conceptualize ITI, it is also necessary to explore the relationship between ITI and technological innovation [36]. An important feature of ITI is that its research scope is broader than that of technological innovation [37]. The industrial entails the integration of companies of the same nature (business scope, company attributes, operating models, etc.), and the relationship of technological innovation between ITI is just like enterprise with industry [38]. Specifically, industrial technology is systematic, large-scale, and universal [39]. Only when a large number of companies in an industry apply a technology can it be called industrial technology. Therefore, ITI can be regarded as a phenomenon of “innovation expansion” in an industry following technological innovation activities [40,41]. Based on the above theory, the concept of ITI can be defined as a complex system with a long span and many participating factors, and a process in which a single or a few companies carry out innovation activities and then spread the innovation to the entire industry.

### Factors affecting the ITI system

The ITI system is a typical artificial system with two main characteristics: the slow progress of “self-organization” and rapid development [42]. The slow progress of self-organization means that in the initial stage of system construction, human-intervention is needed to ensure the normal operation of the system. High-speed development means that system operation consumes a lot of resources; because the system’s initial resources are limited, a large amount of external resources need to be incorporated into the ITI system. In addition, ITI generally comes from national scientific research and industrial transfer, so artificial factors will play an important role in the ITI system [43].

Scholars have proposed that the ITI system is driven by many factors. The driving factors and literature sources are shown in Table 1.

**Table 1:** Factors affecting the ITI system.

Factor	Literature sources
technological breakthroughs	[44]
management level	[27,45]
resource reserves	[26]
agglomeration spillovers	[3]
policy assistance	[46]
industrial transfer	[28]
market demand	[21]

Technological breakthrough refers to the process of new knowledge exploration by research institutes, universities, and other scientific research institutions [47]. A strong technological breakthrough capability can develop products with superior performance, which then improve the product technology level in the market [48]. Insufficient technological breakthrough capability or defects in technology application methods will reduce the rate of increase in knowledge stocks, leading to slow development of ITI [44].

The ITI system involves multiple departments such as enterprises, governments, and individuals. There are complex relationships between departments. The ability to coordinate between different departments and the coordination of competent authorities will affect the development of ITI [49]. The higher the management level of the system, the smoother the communication between departments, and the lower the level of chaos within the system, the less contradiction between departments and the smoother the system development [50]. Conversely, low system management can cause problems and gaps, increase internal disorder within the system, affect the development of ITI, and even cause the ITI system to collapse [27,45].

ITI system operation requires extensive labor and material resources. The amount of resources directly affects whether ITI can continue [51]. Plentiful resources can give a solid foundation for innovation activities and help to improve the quality of innovation. In addition, abundant resource reserves provide powerful support for innovative activities [52]. Conversely, in the absence of support resources, the quality and speed of ITI will be limited, resulting in poor operation of the entire system [26].

Agglomeration spillovers originate from the spatial agglomeration of enterprises, which is accompanied by the intentional or unintentional knowledge diffusion that occurs through inter-enterprise communication [53]. Strong agglomeration spillovers can not only reduce the innovation cost of enterprises but also expand the use scope of and increase demand for innovative products, thus guiding enterprise innovation [54]. Conversely, if there is no agglomeration spillover, the spread of innovation will be inhibited, market demand for innovative products will weaken, and corporate profits will ultimately decline. In addition, weaker agglomeration spillovers can cause the standardization process to slow down, which is not conducive to the development of ITI systems [3].

The government's policy assistance is artificial intervention in the performance of ITI. The establishment of an ITI system is inseparable from the guidance and assistance of the government. Besides providing tax and financial assistance to companies in the ITI system, the government will also adopt policies such as talent introduction [55]. Because ITI is generally derived from national scientific and technological research, it has obvious policy orientation [56]. Some types of ITI will be significantly accelerated by national resource support and policy orientation. Conversely, other types of ITI that do not receive policy support are relatively slow to develop [46].

Industrial transfer from developed regions is an important driver of ITI [57]. Developed areas are far away from raw material supply areas, and labor costs and environmental carrying capacity are approaching their limits [58]. Therefore, the transfer of industry to other areas can strengthen industrial scale [59] and provide more room for development, thus enhancing quality and competitiveness. It can also promote the optimal allocation of resources and regional economic layout, and ultimately the common development of ITI [28].

Market demand is the focus of supply-side reform and an important driver of ITI. Enterprises will allocate more resources to support innovation and promote the steady development of the ITI system [60]. Weak market demands will cause the company cannot to recover the research cost, thus affecting the innovation environment and the sustainable development of the ITI system [21].

## The foundation of the pioneering method: The link between ITI and systems thinking

Systems thinking can identify problems within the system and solve the root cause of these problems through a multi-disciplinary “frame” [61]. At present in China, individuals, enterprises, and even the government are advocating technological innovation and system development, which means that future technological innovation presents many challenges [62]. Relying on a simple linear perspective is not sufficient to resolve these challenges; it is also necessary to adopt nonlinear systems thinking and to explore thinking and decision-making from a perspective more in line with the laws of nature [63]. Systems thinking pay more attention to the role of causality in nature and society [64]. Under certain conditions of uncertainty and long-term relevance, systems thinking can determine the relevance of system elements through analyzing the existing whole, so as to realize the “observation of elements and their mutual relations” and “find the law of change instead of static slices” [65]. Therefore, systems thinking is a method that can help people to “learn to take effective decisions” and develop new ways of thinking and action guidelines [66]. Compared with the traditional linear method of identifying “specific defects and repairing them,” repairs under systems thinking will improve the unitary consequences caused by “systemic myopia” [67]. Modifications under systems thinking will prompt changes in the “now” to cause changes in the “later,” thereby filling the deficiencies caused by systemic myopia [68].

The system complexity of ITI [3] is manifested in three aspects: nonlinearity [69], long-range correlation [70], and uncertainty [71]. The concept of nonlinearity comes from modern mathematics [72] and refers to the absence of a linear relationship between two variables. In the ITI system, nonlinearity is mainly reflected in uncertainty over the correlations between various factors in the system. The ITI process is affected by uncontrollable factors, which cause nonlinearity that is mainly reflected in the randomness of the ITI direction and the uncertainty of ITI outcomes. These nonlinear relationships reflect the high complexity of the ITI system.

Long-range correlation originates from the “butterfly effect” [73], which refer to the phenomenon of a final result being extremely changed by a minimal change in one or more influencing factors. In the ITI system, long-range correlation is mainly manifested by amplification in the transmission chain. Each element in the ITI interacts with other elements to ultimately form systemic relationships. Any change in the system will bring about changes in the entire system, and small changes will be gradually amplified, resulting in subversive changes in the final outcomes [74]. Changes in the influencing factors in the ITI system will significantly impact on the evolution of ITI. Therefore, long-range correlation also reflects the systems thinking view of ITI.

Uncertainty [75] is a physical concept that refers to a state in which the time, direction, and frequency of changes in system elements are unpredictable. Complex systems exhibit an unbalanced state resulting from long-range correlation and nonlinearity. The evolution of the ITI system stems from the mutual integration of companies, governments, individuals, research units, and other factors based on the system framework [76]. In addition, factors outside the system boundary (people, technology, etc.) are constantly being imported into the system, and the system environment is also changing [77]. The ITI system is also constantly self-adjusting and developing, changing the existing system framework and operating mechanism, and promoting the further development of the ITI system. Therefore, uncertainty is also a systemic manifestation of ITI.

**Fig 1:**
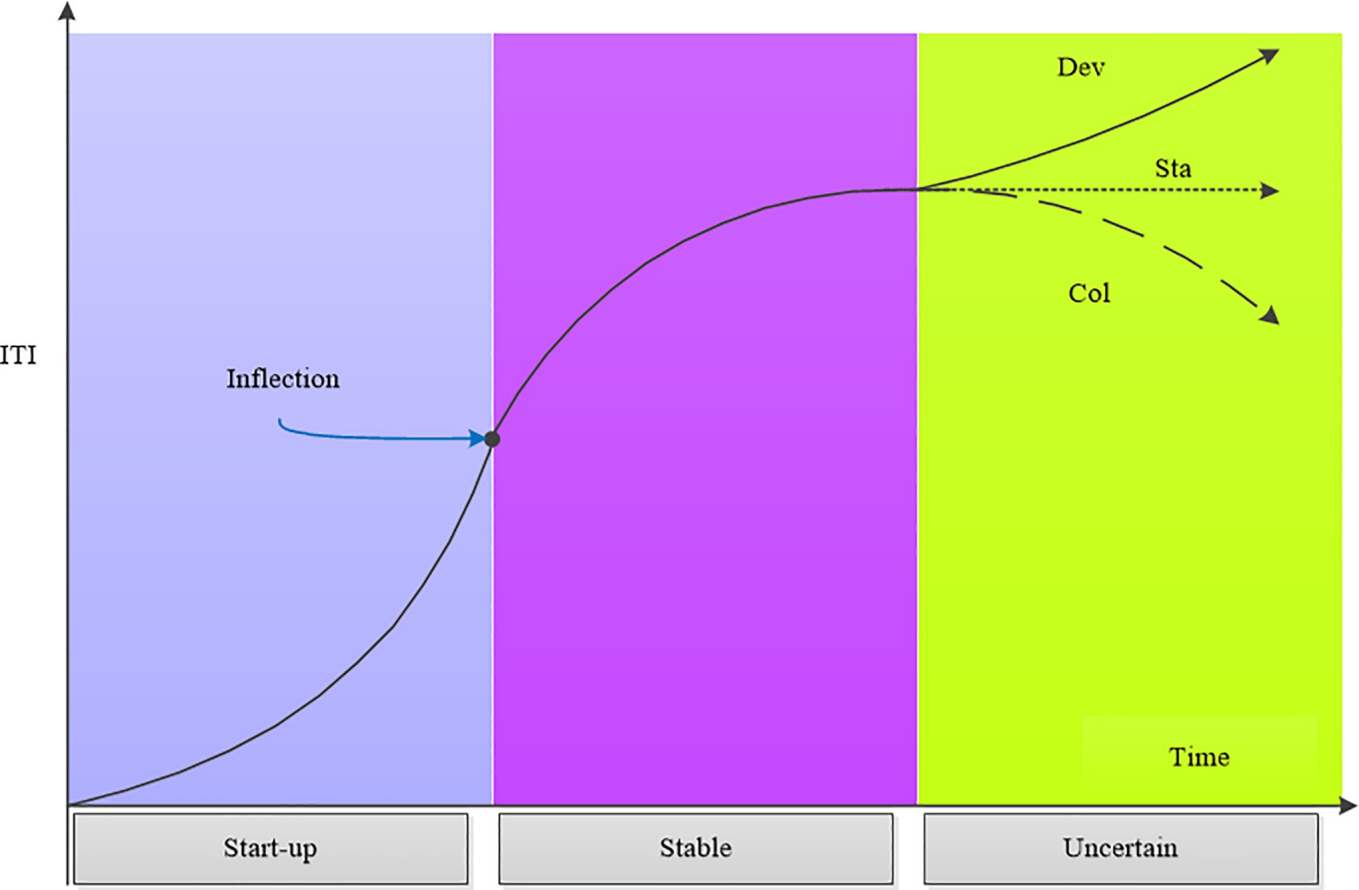
The evolution trends of the ITI system.

The ITI system development model can be generally defined as an approximate S-shaped curve [3] (Fig 1). This curve represents the changes in the ITI system at different stages over time. Following its initiation, ITI initially develops quickly. Although the degree of development needs to be improved, the ITI development mechanism dominates in the start-up phase. As the ITI system develops, some inhibitors increasingly emerge, which slow down the development of ITI, even if the degree of development is high. Based on this theory, the ITI system goes through two stages respectively dominated by positive promotion and negative inhibition [13].

The rapid exponential development in the early stages is unsustainable, and so slows down over time, before growth eventually becomes steady under equilibrium constraints. The inflection point indicates where the second derivative of the curve is zero, marking the deceleration of ITI development. There are three types of ITI in the future: “Development” (Dev), “Stagnation” (Sta), and “Collapse” (Col). In the development state of “Stagnation,” the ITI system is unable to supply innovation and development, and so cannot achieve sustainable development. Similarly, the lack of innovation support will cause innovation communication to cease, resulting in system downtime and eventually collapse. Even if this “Collapse” state is temporary, it cannot produce sustainable development. Only the “Development” of ITI meets the requirements for sustainable development. Under this state, a new round of innovation and development can be carried out.

## Research methodology

### System archetypes

The tools for managing complexity created by systems thinking are very rich, and one of the important tools is the system archetype [78,79]. Rather than describing specific problems, a system archetype summarizes the common problems of a series of phenomena, and has a variety of forms and a clear structure [80]. The passage of time drives the development of the archetype, which exhibits different behaviors over time. Based on the basic structure of the system archetype, high-quality strategies are defined to deal with the identified bottlenecks [81]. The function of the system archetype is to provide solutions to the dynamic changes in complex systems. This method embodies the decision makers’ judgment on the structure and behavior of a specific complex system, and is convenient for communicating the problems existing in the system and for leveraging interventions to change it [82]. The system archetype reveals the underlying structure of the ITI system, making it possible to predict current problems in the ITI system and propose ways to solve them [83].

As one of the keys to breaking bottlenecks, the system archetype is helpful for understanding the ITI system and promoting technological innovation. The system archetype involves deeper thinking for understanding and solving problems. The approach is not to “solve current problems immediately” but rather to solve “major challenges in the future” [84]. This research will use systems thinking to analyze the operation of ITI, use the system archetype to analyze the causality and system loops of the factors affecting ITI, and explain the “now” and “future” of different factors. The system archetypes we use are all general system archetypes that can explain the dynamic changes of ITI system elements. The proposed system archetypes can also help decision makers make decisions that meet their own needs.

### System element constraints

During the evolution of the organizational structure of a complex system, each originally dispersed element or subsystem will continuously interact within the system [2], and which defines “constraint coordination” in systems science [85]. The influence path of one element on another element in the system is called "constraint" [86].

**Fig 2:**
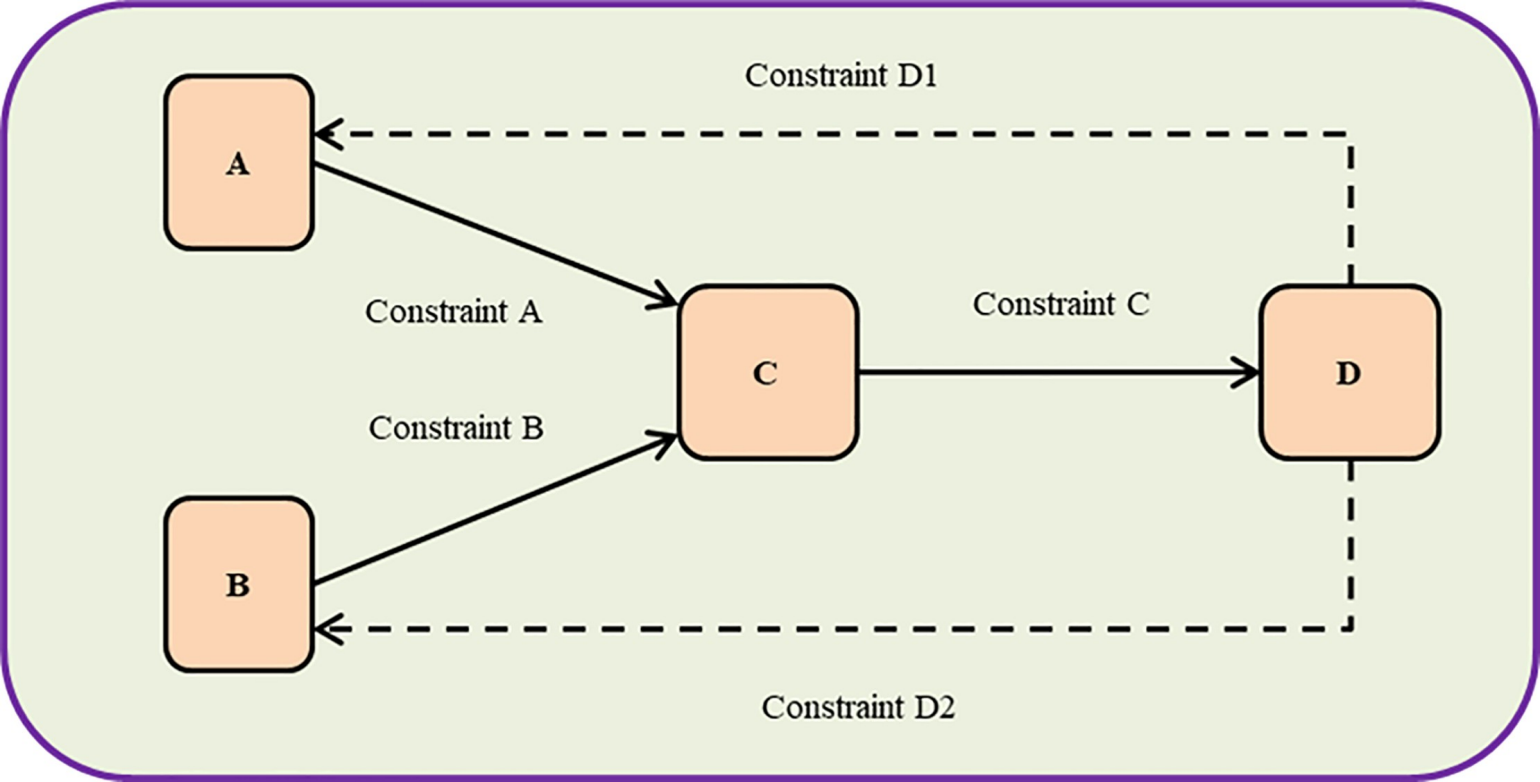
System elements and constraints. *Note: Constraint D is a coordinating constraint.

Based on systems science, this study defines system element constraints as the effects that cause the evolution of system elements to produce certain changes, specifically referring to the interaction between system elements. It should be noted that there is constraint coordination between system elements. System elements are not only recipients but also producers of constraints as shown in Fig 2.

Constraints A and B are the system elements that constrain element C, which we define as “input constraints”; constraint C is the number of element C constraints on element D, which we define as “output constraints.” The interaction between system elements is no superimposed and nonlinear. The number of system element constraints can be used as one indicator to judge the core elements in complex systems. The calculation of constraints is as follows:

constraints=sum(inputconstraints,outputconstraints) (1)

## Results and discussion

In this section, the study combines the complexity characteristics of the ITI system and the drivers model to extract and analyze the operating mechanism of six archetypes: “Limit to Growth,” “Success to the Successful,” “Tragedy of the Commons,” “Fixes that Fail,” “Accidental Adversaries,” and “Shifting the Burden” [87]. Based on the status quo of China’s industrial development, this study explores the impact of the above-mentioned driving factors on the ITI system and proposes a leverage solution for each factor.

### Technological breakthrough: “Limit to Growth” archetype

**Fig 3:**
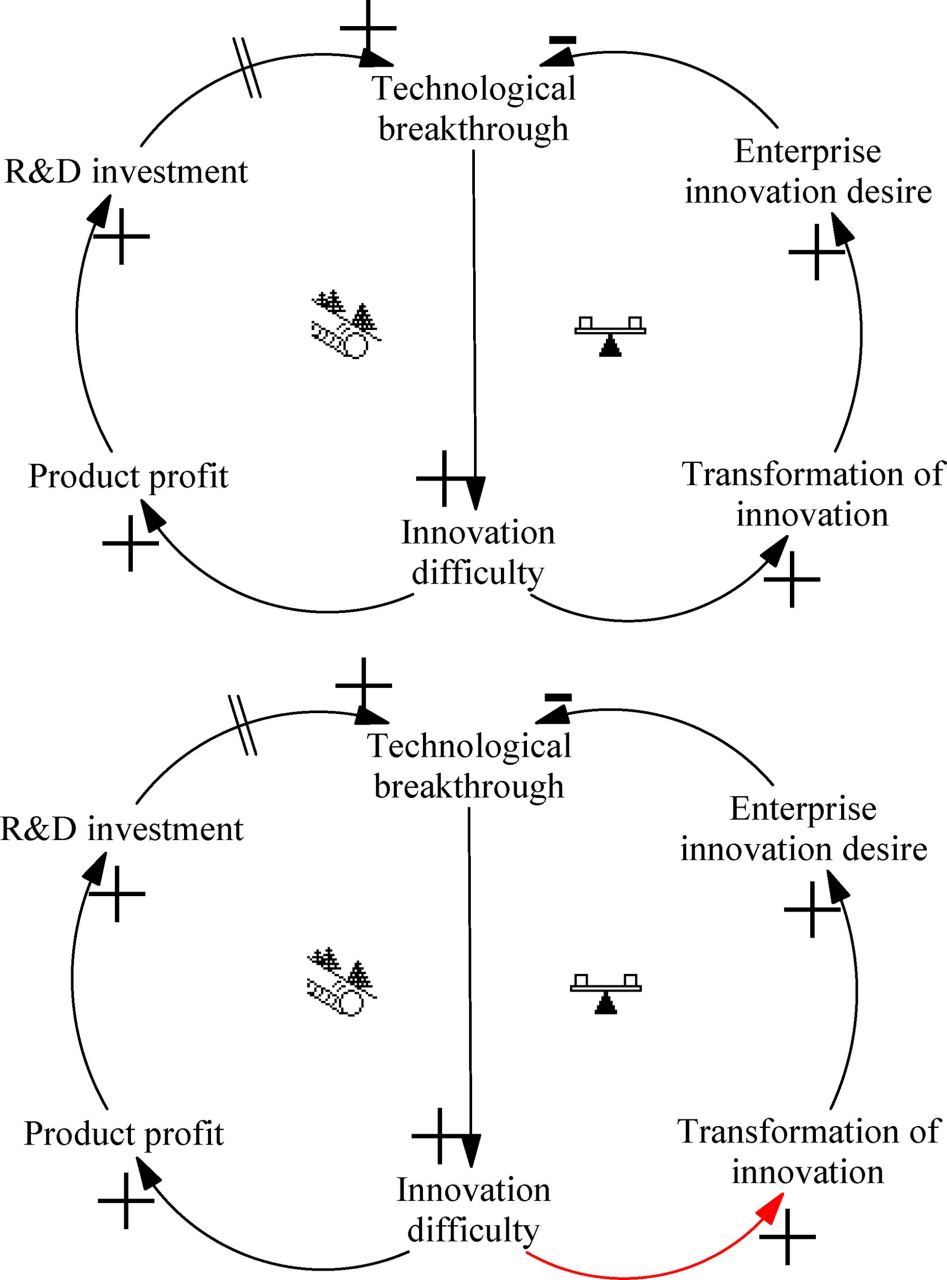
Archetype of technological breakthrough.

The “Limit to Growth” archetype of technological breakthrough describes the negative outcomes that accumulate from striving to achieve higher levels of innovation, which can lead to system sluggishness, eventually slowing system development and potentially causing system collapse. The structure has a positive feedback cycling loop and a corresponding limiting loop, as shown in Fig 3.

As a component of the ITI system, companies directly determine the development of the ITI system through their behavior. Rational companies want more profits, and more difficult innovations will increase the profitability of innovative products. Striving for excess profit, enterprises will increase the inputs of funds and personnel into R&D, and continue to support technological breakthroughs to obtain lucrative product profits. This forms a positive cycling loop, and the elements in the loop continue to strengthen one another’s influence, promoting the rapid development of technological breakthroughs. However, with the continuous development of technological breakthroughs, the difficulty of transforming them into innovation achievements also rises, leading to increasing costs of innovative production. Excessive innovation costs will inhibit companies’ willingness to innovate, and ultimately lead to a decline in technological breakthroughs. Especially when a large number of innovations cannot be turned into products, extensive resources will be wasted and the sustainable development of the ITI system will be hindered.

Leverage solution (Fig 3B): Do not blindly strengthen the forward loop but instead analyze the existing negative feedback loop to break the bottleneck. Bottleneck in this archetype is the ability to transform scientific and technological achievements; only by enhancing this ability and ensuring that enterprises can recover the cost of innovation can the development of the ITI system be promoted. In other words, the development of the right side of the loop should be improved, rather than blindly strengthening the left side. China has a large number of scientific researchers and scientific research achievements, but the transformation of scientific research results into innovative products still needs to be improved, in terms of both quantity and quality. China should promote the combination of research and production, establish an incubation system for innovation results, and improve the transformation of innovation results.

### Management level: “Success to the Successful” archetype

**Fig 4:**
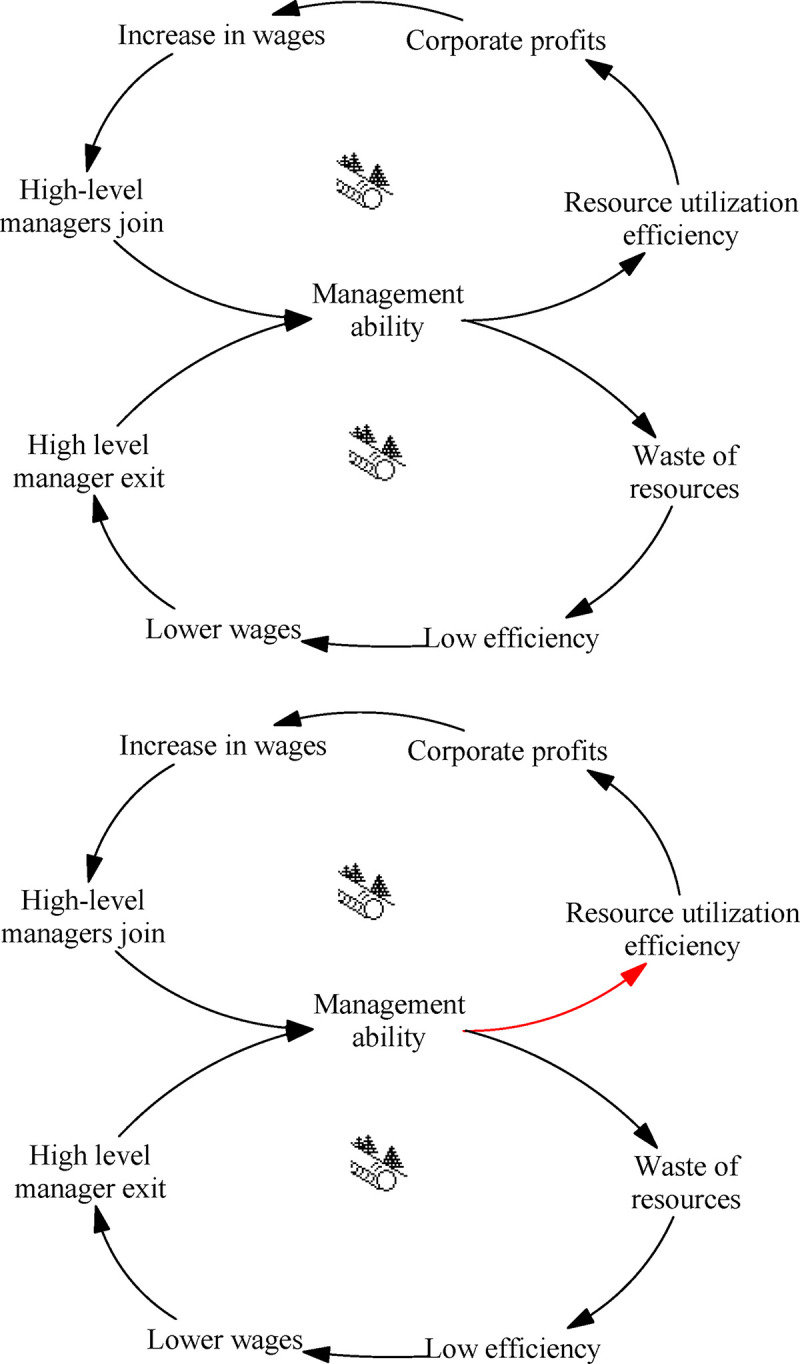
Archetype of management level.

The “Success to the Successful” archetype of management level describes the two-loop feedback structure, which is a contrast system. In the initial stage of system operation, the difference between the two loops is small, but when differences appear between specific factors in the loop, a negative snowball effect occurs and also spreads to the other loop, causing strengths to grow stronger and weaknesses to intensify in the double-loop system. This is not conducive to the overall development of the ITI system. Fig 4 depicts this process.

There is large heterogeneity in the management level of innovation in different Chinese enterprises. Higher management ability can increase resource utilization efficiency, thereby reducing costs and allowing companies to make more profits. Conversely, companies will be unable to provide competitive salaries, resulting in the exit of high-level managers, thus further reducing the management level. This phenomenon is not conducive to the general development of the ITI system.

Leverage solution (Fig 4B): It is necessary to proceed from the system, rather than separating the two loops, rationally allocate resources, establish a talent introduction plan, and promote the development of enterprise management. Besides training senior management to improve managerial ability, it is also necessary to promote talent exchanges in different regions, absorb high-level talents,. Although China has a large economic aggregate, the average salary is lower and the ability to attract high-level talents is limited, compared with developed countries. In addition, China has fewer top universities compared with countries such as the United States. There is still a large amount of brain drain every year. China should strengthen the introduction of top talents and optimize the talent structure.

### Resource reserve: “Tragedy of the Commons” archetype

**Fig 5:**
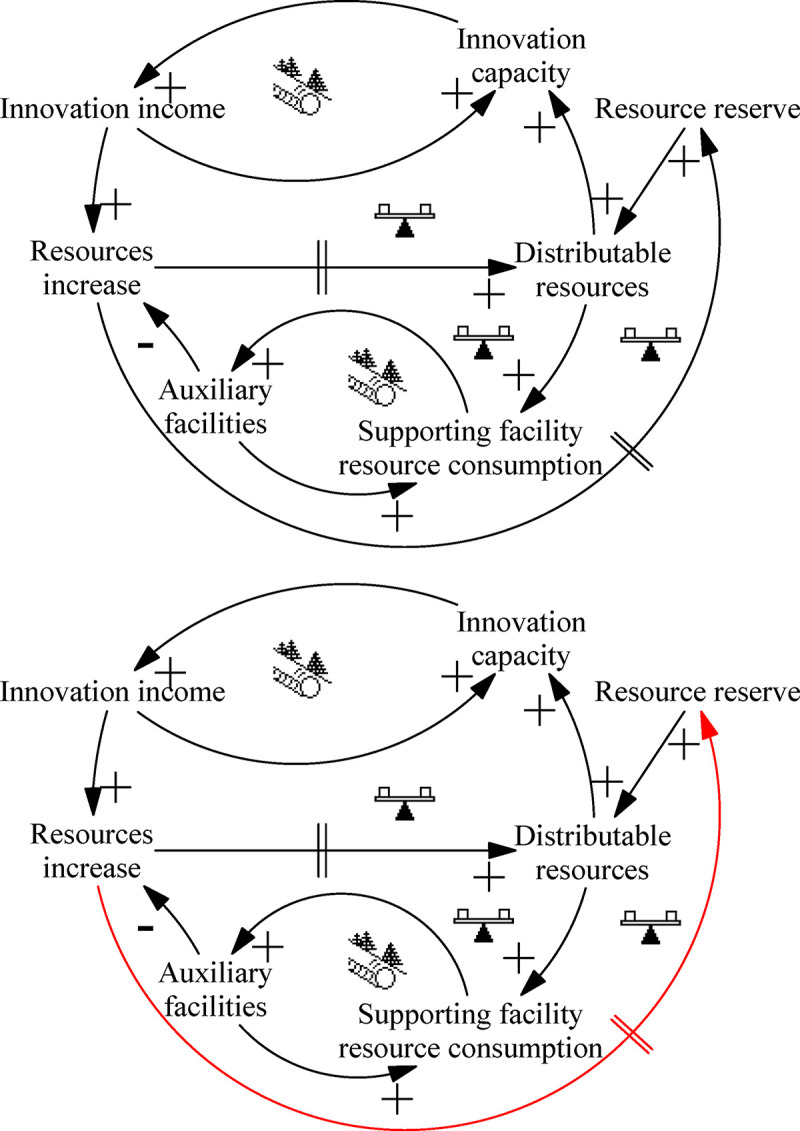
Archetype of resource reserves.

The “Tragedy of the Commons” archetype of resource reserves describes the excessive use of and competition between system elements over limited public resources. In the initial stage of system development, the total amount of resources is relatively abundant, and each subsystem can acquire the resources needed for its own development, enabling high-speed development. However, at a certain stage, the rapid development of the system leads to the huge consumption of public resources, and it can no longer be effectively supplemented in the short term. At this point, each subsystem competes for resources to meet its own development, resulting in a decline in resource reserves, and the system will collapse as resources are exhausted. Fig 5 depicts this process.

The operation of the ITI system requires multi-sectoral collaboration. To facilitate understanding of the “Tragedy of the Commons” archetype, we assume a situation where there is only one innovative subsystem and one supporting facility subsystem. The two departments allocate available resources according to their own conditions. In the initial stage of the ITI system, the depth and scale of innovation and the corresponding supporting facilities are relatively low; resource consumption during this period is limited and can meet the needs of both departments. As the scope and depth of innovation gradually grow, the requirements for supporting facilities also gradually increase. To ensure the normal operation of the system, the amount of resources consumed by each subsystem rises sharply. The innovative subsystem and supporting facility subsystem then compete for limited resources to support their own development. The result is either innovation with no supporting facilities or supporting facilities with no innovative R&D, which ultimately leads to a decline in the efficiency of the ITI system.

Leverage solution (Fig 5B): Accelerate system resource regeneration to prevent the phenomenon of subsystems maliciously competing for resources. The total amount of resources is limited in the short term, and increasing resources also takes a certain amount of time. Therefore, the cycle of resource regeneration plays a decisive role in system operation. By strengthening management methods, the efficiency of resource utilization will be improved and the delay will be shortened, thereby achieving a relative increase in the total amount of available resources. When the innovation department and the supporting facilities department develop at the same or similar speed, ITI can be optimized. By contrast, when there is a mismatch between the rates of development of these subsystems, the level of innovation will decline, thereby inhibiting China’s sustainable development.

### Agglomeration spillovers: “Fixes that Fail” archetype

**Fig 6:**
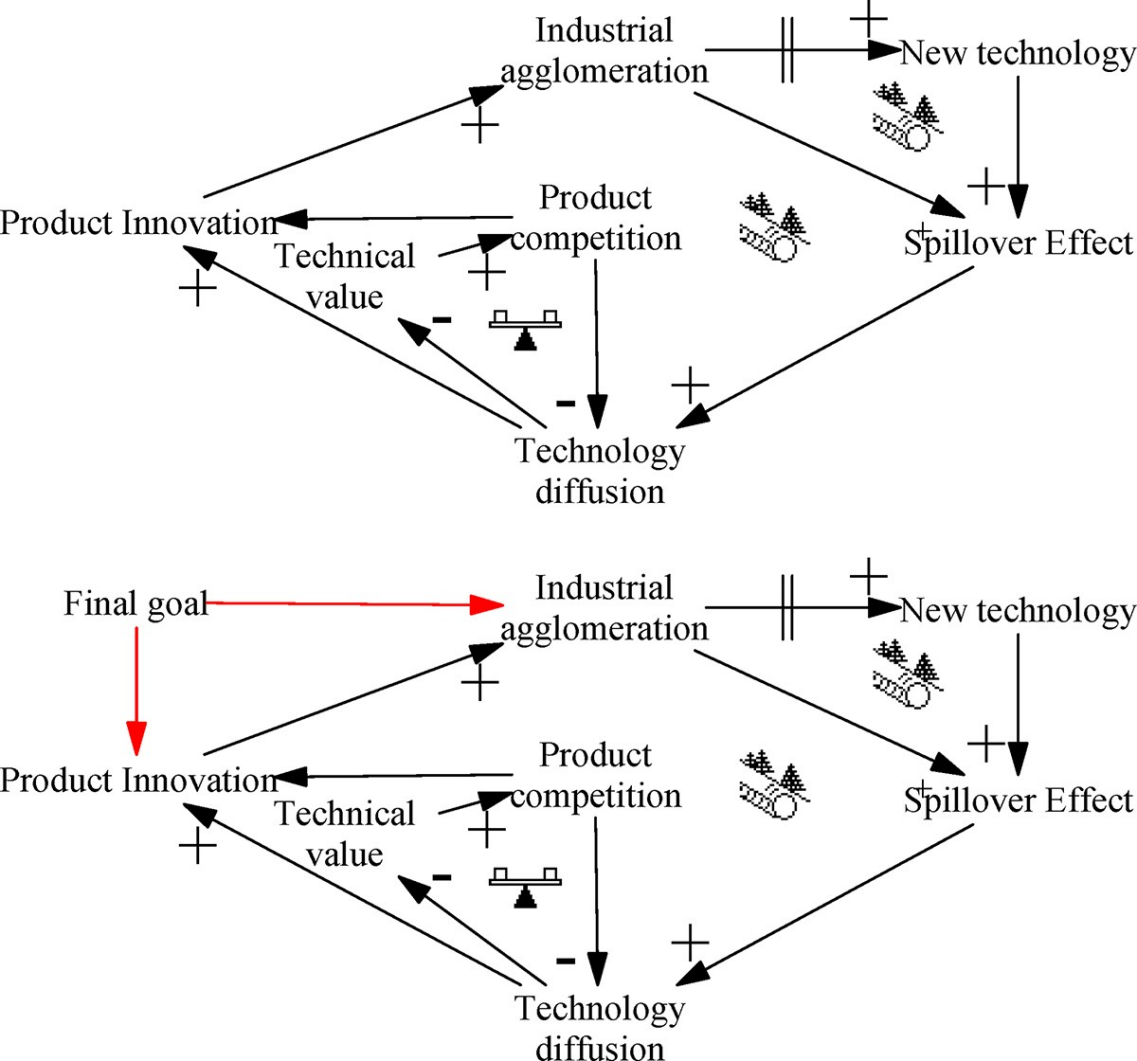
Archetype of agglomeration spillovers.

The “Fixes that Fail” archetype of agglomeration spillovers describes when situation where emergency measures to modify and correct serious system errors or deviations in the short term lead to unexpected negative effects. If short-term means are overused, the negative effects will slowly accumulate, eventually leading to unintended consequences. Fig 6 depicts this process.

Industrial cluster spillovers can complete the diffusion of technology in a short time period, after which different companies will produce products with certain heterogeneity and gain innovation profits. However, after the technology spreads, the technical value of innovative products will be reduced. Especially for leading enterprises, high R&D expenses cannot be fully recovered, leaving a lack of funds for the next round of R&D. Other companies prefer to imitate and are reluctant to carry out their own R&D, resulting in the falling quality of innovation, and ultimately preventing the sustainable development of ITI.

Leverage solution (Fig 6B): Establish and address the underlying, fundamental problem, reduce the use of short-term measures, and avoid the congestion of hidden accumulation. As shown in Fig 6, the fundamental problem is establishing an ITI system. Agglomeration spillovers are only a means, not the end. It is necessary to protect the independent innovation results of enterprises, encourage enterprises to carry out R&D of new technologies, curb the effects of negative loops, and avoid vicious competition. China should take a long-term approach in dealing with agglomeration spillovers, set up R&D units in industrial zones, and strengthen cooperation between R&D departments and production companies.

### Policy assistance: “Accidental Adversaries” archetype

**Fig 7:**
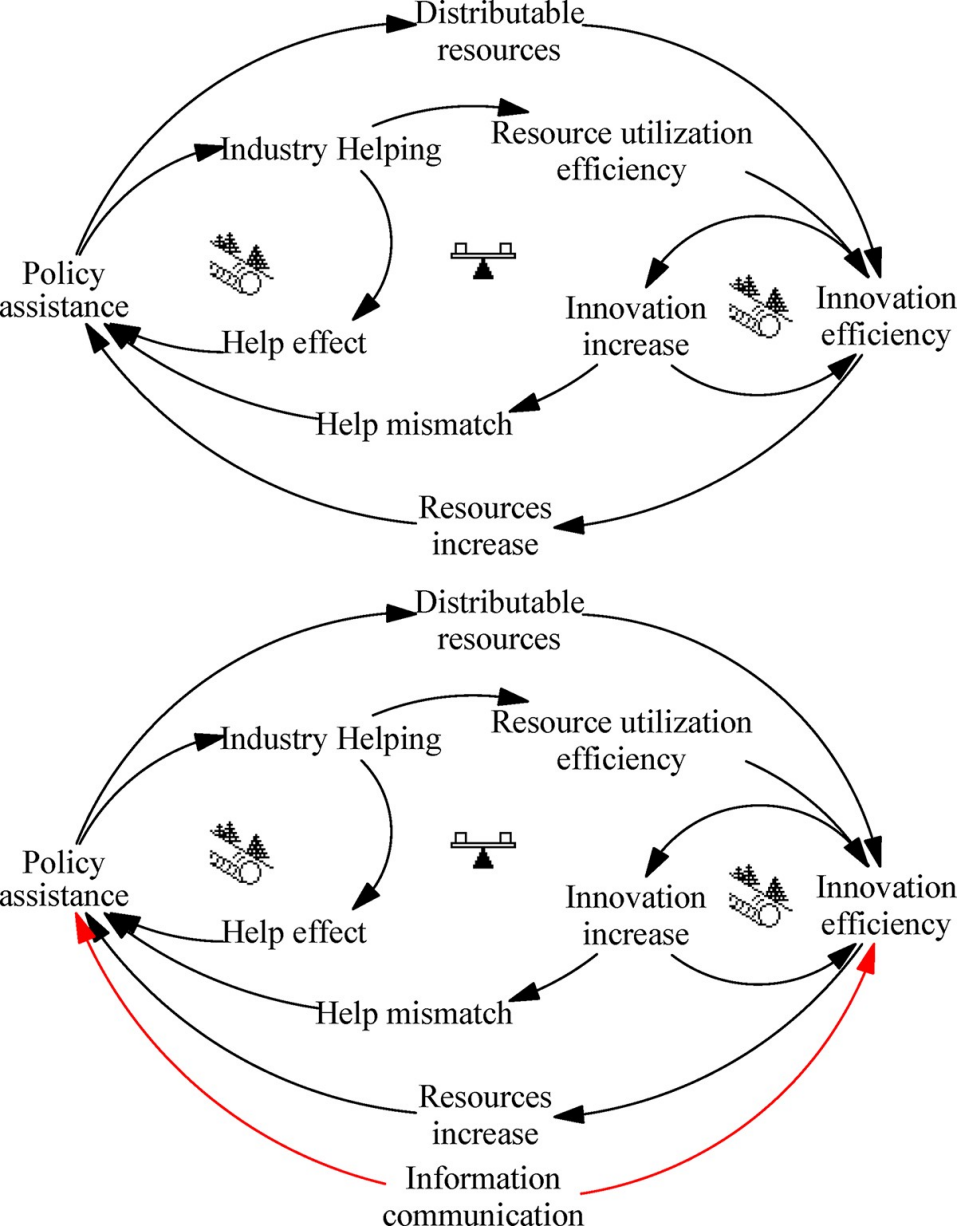
Archetype of policy assistance.

The “Accidental Adversaries” archetype of policy assistance describes the situation where subsystems that should complement each other or at least work together come to hinder each other’s development, eventually resulting in cooperation failure. Although both subsystems cooperate toward the same goal, the operation of one will unexpectedly hinder the operation of the other, thus inhibiting its development; as feedback, the affected subsystem will interfere with the operation of other subsystem. Through the accumulation of multiple negative influences between the two subsystems, the entire system becomes unstable and system operation tends to stagnate. Fig 7 depicts this process.

Policy assistance can increase the available resources, thus enhancing the efficiency of technological innovation. In turn, more efficient technological innovation contributes to the implementation of policy assistance, and both the policy assistance and technological innovation subsystems run smoothly. However, each subsystem maybe ignores the other’s needs and status. The imbalanced distribution of funds by policy assistance will increase the difficulty of resource acquisition by less-supported departments and curb the efficiency of technological innovation. Conversely, the increase in innovation will break through the original scope of assistance and reach areas not covered by the original policies, resulting in inefficient policy support. Although each subsystem can recognize the other’s unintended influence on itself, each believes that the other should take the lead in making changes, eventually leading to a breakdown in cooperation.

Leverage solution (Fig 7B): Communication should be strengthened to build a deep understanding of the other subsystem’s needs and promote the coordinated development of subsystems. Policy assistance should pay attention to understand the latest needs of the innovation department, and actively adjust policy measures. For its part, the innovation department needs to provide timely feedback on its own situation, so that the policy department can promptly modify policy assistance and promote the coordinated development of ITI. A large number of enterprises believe that there is still a substantial misalignment between government policies and the needs of enterprises [88]. Government departments should strengthen on-the-spot investigations, understand the actual needs of enterprises, help enterprises achieve technological progress, and promote ITI.

### Industrial transfer: “Shifting the Burden” archetype

**Fig 8:**
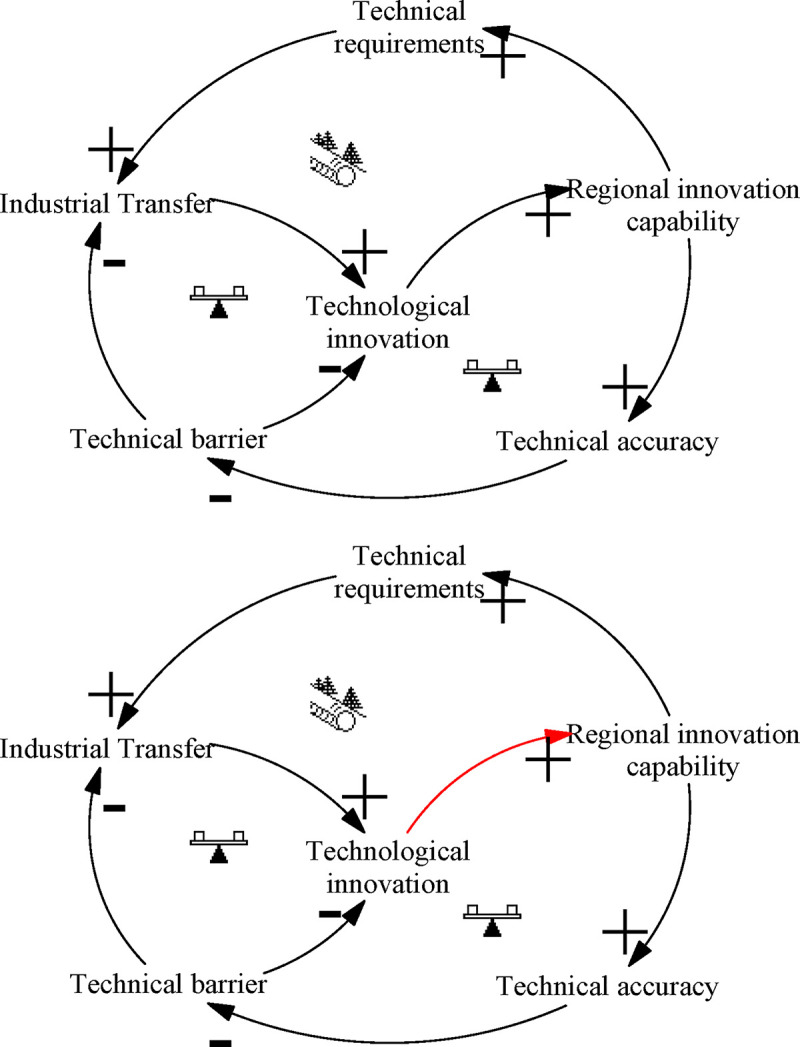
Archetype of industrial transfer.

The “Shifting the Burden” archetype of industrial transfer describes the situation where short-term goals of the system are pursued or short-term measures deployed without thoroughly exploring the nature of problems. The shortcomings of short-term solutions can result in the accumulation of negative effects, which may eventually cause serious losses. Fig 8 depicts this process.

Industrial transfer significantly boosts the innovation capacity of underdeveloped regions, and the resulting technological demand further strengthens industrial transfer, eventually forming a closed positive loop. However, industrial transfer is mostly of mature technology, with high technical precision and narrow technological application. There is also weak technology spillover capability, which raises technical barriers and leads to innovation extrusion. In addition, due to limited resources, managers may be myopic or pay too much attention to short-term benefits in the next round of industrial transfer, and so neglect the introduction of future innovation industries. Although in the short term their approach will significantly improve regional innovation capabilities, from the long-term perspective it will lead to a narrower spectrum of technology in industrial clusters and increasingly homogenized industry, unable to adapt to market complexity, resulting in falling innovation efficiency.

Leverage solution (Fig 8B): Identify the nature and roots of the problem and apply long-term solutions (similar to the “Fixes that Fail” archetype”). Industrial transfer is only a means to quickly promote ITI in the short term, rather than the final form of the results of the ITI system. After undertaking industrial transfer, China should strive to enhance the regional innovation carrying capacity so as to assimilate deeper industrial technologies. However, China must not blindly undertake industrial transfer. To attract foreign investment, China has set up a large number of preferential conditions for industrial transfer, and has upgraded local industrial technology in a short period of time. However, in the absence of preferential policies, the local industrial park will be abandoned, resulting in a huge waste of resources. China’s industrial transfer policy should be adjusted to ensure the self-organization of the ITI system, with a view to establishing a long-term ITI development mechanism.

### Market demand: “Limit to Growth” archetype

**Fig 9:**
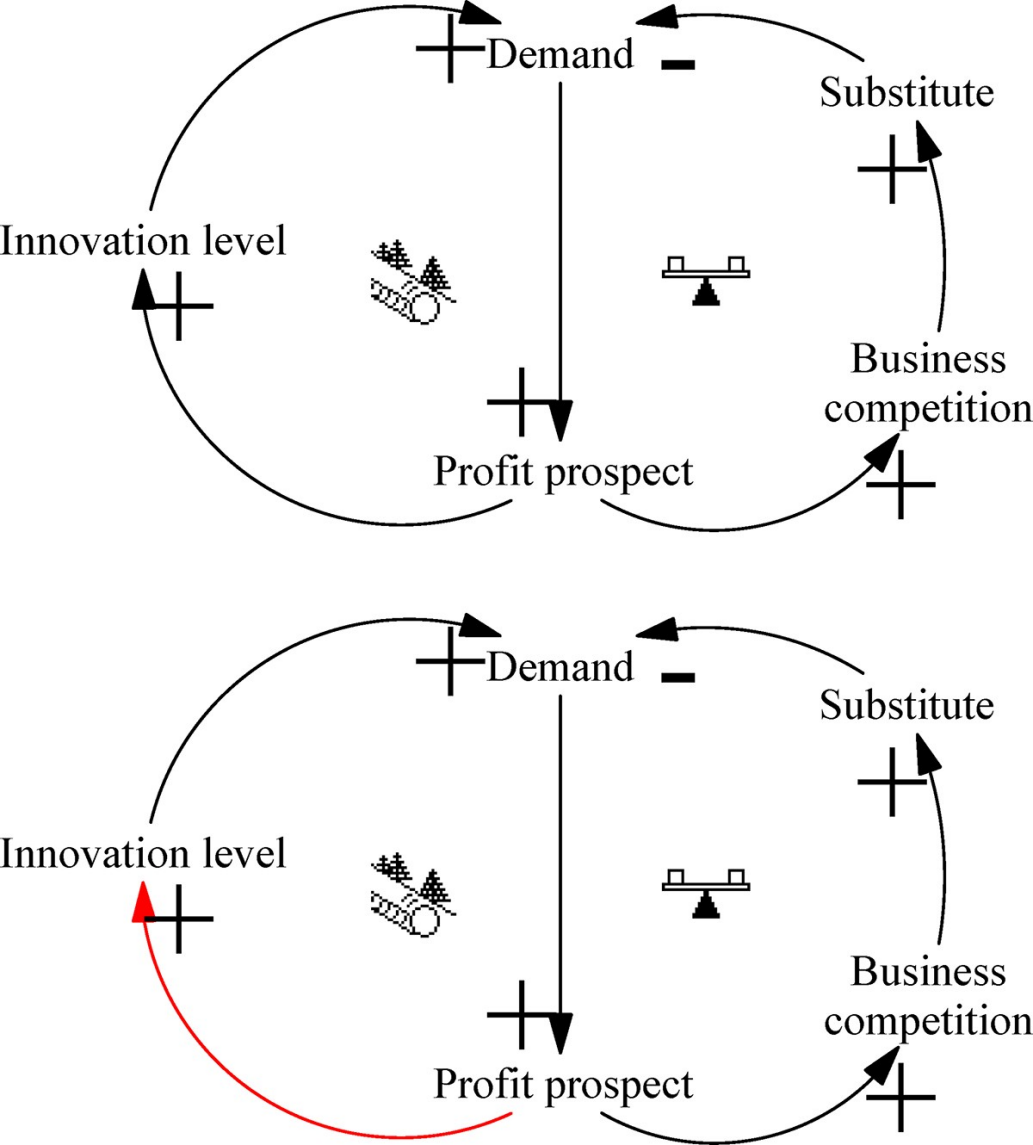
Archetype of market demand.

The “Limit to Growth” archetype of market demand describes a similar model of loops to that of the archetype of technological breakthrough: it is described as facilitating the development of the loop, resulting in the accumulation of the negative limiting loop, which ultimately causes the system to decline after a certain period of development. Fig 9 depicts this process.

In the early stages, a large number of innovative products are demanded, offering considerable profit prospects. To maintain the existing profit level, enterprises continue to strengthen investment in innovation, research and develop new innovative products, and obtain greater profits. Repetition of this cycle promotes the rapid development of the ITI system. At the same time, other competitive enterprises compete for innovative R&D and product imitation in order to survive, and alternative products will be introduced to meet market demand. The increase in product supply capacity directly leads to a decline in equilibrium prices, causing profits to fall sharply. In later stages, it is also possible for vicious competition to ensue among enterprises, detrimentally impacting the innovation environment and harming the sustainable development of ITI.

Leverage solution (Fig 9B): Control the early stages of the suppression loop and enhance the boost loop. The “Limit to Growth” archetype applies to both market demand and technological breakthroughs, but the leverage solutions differ completely. Suppressing the negative loop will not help the development of the system. This archetype needs to be regulated in the early stage of the system to prevent the ITI system from crashing. In other words, when there are no alternative products, investment in scientific research should be strengthened and innovative products should be developed. It is necessary to guarantee the source of corporate profits and avoid the problems of falling profits caused by negative loops. China has long been in middle and low positions of the industrial chain, with dominant roles for imitation, learning, and catch-up industries. The main problem with China’s ITI systems is their inability to meet the innovation demand of Chinese customers. Market demand and demand dynamics should be accurately identified to produce innovative products that meet market needs. At the same time, innovative product has a direct orientation to guide market demand, improve the level of innovation, and promote the development of ITI systems.

### ITI system integration model

By combining Figs 3–9 and performing a path analysis [89,90] to eliminate redundant constraints, we obtain the ITI system integration model, as shown in Fig 10.

**Fig 10:**
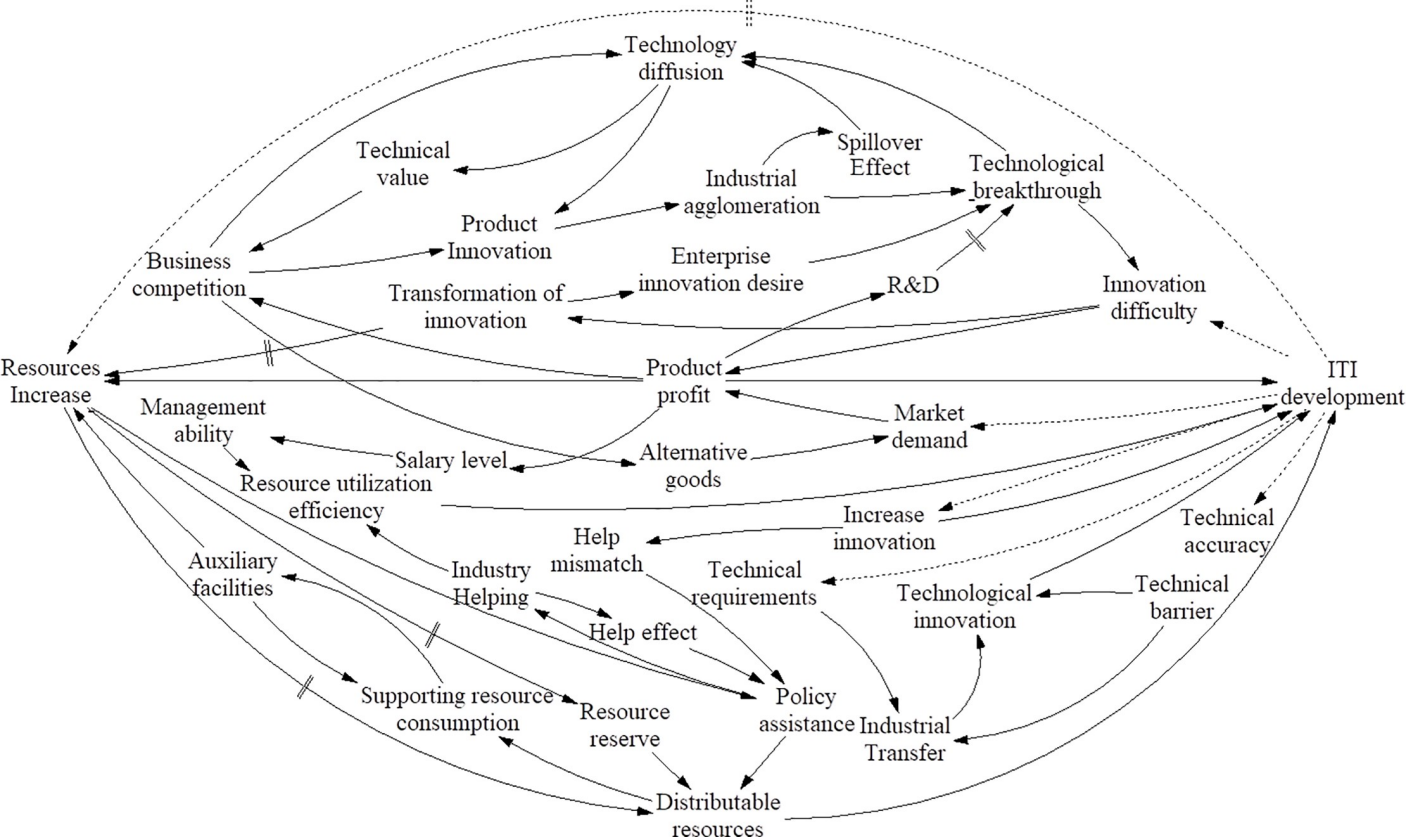
ITI system integration model.

After analyzing the system element constraints, we identify the constraints of each element in the IT system. As shown in Table 2, “product profit” and “resource increases” each have seven constraints; “technological breakthrough,” “technology diffusion,” “disposable resources,” and “enterprise competition” each have five constraints; and absolute bondage has one constraint.

**Table 2:** The constraints of elements.

Element	In constraints	Out constraints	Constraints
Product profit	2	5	7
Resources Increase	4	3	7
Technology diffusion	3	2	5
Business competition	2	3	5
Distributable resources	3	2	5
Technological breakthrough	3	2	5
Innovation difficulty	2	2	4
Resource utilization efficiency	2	1	3
Auxiliary facilities	1	2	3
Industry Helping	1	2	3
Supporting resource consumption	2	1	3
Product Innovation	2	1	3
Transformation of innovation	1	2	3
Technological innovation	2	1	3
Market demand	2	1	3
Industrial agglomeration	1	2	3
Increase in innovation	1	2	3
Industrial Transfer	2	1	3
Technical barrier	0	2	2
Alternative goods	1	1	2
Management ability	1	1	2
Technical value	1	1	2
Salary level	1	1	2
Help mismatch	1	1	2
Help effect	1	1	2
Resource reserve	1	1	2
Enterprise innovation desire	1	1	2
Spillover Effect	1	1	2
R&D expenses, personnel	1	1	2
Technical requirements	1	1	2

From the above (Table 2), it can be concluded that the core elements of the ITI system are product profit and resource increase. Product profit is needed to build a strong innovation ecosystem, which includes not only innovation policies but also the innovation environment, innovation awareness, and all-round improvement of innovation capabilities. In addition, China should pay attention to innovation facilities and market changes, so as to avoid the challenge of the “Tragedy of the Commons” archetype. Because resource reserves are difficult to change, resource increase depends on the rate of resource regeneration, but this does not mean that resource reserves are irrelevant. In its early stage, the ITI system needs to absorb a large amount of external resources because self-organization develops slowly. Therefore, in the initial stage of system construction, it is very important to provide a resource guarantee. In the middle and late stages of ITI system construction, efforts should be made to build an innovation ecosystem to help the completion of self-organization, shorten the resource regeneration cycle, and promote the development of ITI systems.

## Conclusions

The impact of innovation on the economic development of countries around the world has been recognized, and governments are promoting innovative activities to progress toward sustainable development. However, lasting ITI development requires a comprehensive understanding of the complexities associated with ITI systems, and controlling the key factors will drive the development or collapse of ITI systems. To better understand the interaction between structure and behavior, we propose using systems thinking to analyze the dynamic complexity of the ITI system. This strategy can be used to efficiently handle the steady operation of ITI systems, reduce resource waste, and solve major challenges in the future.

Based on systems thinking, this study decomposes complex factors into external and internal factors, and constructs six archetypes: “Limit to Growth,” “Success to the Successful,” “Tragedy of the Commons,” “Fixes that Fail,” “Accidental Adversaries,” “and Shifting the Burden.” It thereby helps to capture the dynamic complexity and scalability of ITI systems. This approach allows stakeholders to better understand the current state of the ITI system and how it is coordinated. More importantly, by using systems thinking methods, decision makers can make long-term predictions across stages and recognize the disruption of plans by sporadic factors. This should help policy makers to build a systemic intervention framework to promote the sustainable development of the ITI system.

Finally, by constructing an ITI system integration model and identifying the constraints of all factors, this study promotes in-depth understanding of the development path of the ITI system. Specifically, China should continue to reduce government intervention and promote the self-organization of the ITI system. It also needs to construct a reasonable resource allocation system for innovative systems in order to achieve sustainable development of ITI systems.
